# Understanding Pediatric Patient Experiences with Urotherapy Tools: Qualitative Focus Group Study

**DOI:** 10.2196/79142

**Published:** 2025-10-16

**Authors:** Lola Bladt, Anka Nieuwhof-Leppink, Rose-Farah Blomme, Jiri Vermeulen, Alexandra Vermandel, Gunter De Win, Lukas Van Campenhout

**Affiliations:** 1 Department of Product Development, Faculty of Design Sciences, University of Antwerp, Paardenmarkt 90/94, Antwerp, 2000, Belgium, 32 497848014; 2 Department of Research and Development, Minze Health NV, Antwerp, Belgium; 3 Department of Urotherapy, Psychology and Urology, Wilhelmina Children’s Hospital, University Medical Center Utrecht, Utrecht, The Netherlands; 4 Department of Rehabilitation Sciences and Physiotherapy, Faculty of Medicine and Health Sciences, University of Antwerp, Antwerp, Belgium; 5 Department of Urology, University Hospital Antwerp, Edegem, Belgium; 6 Antwerp Surgical Training, Anatomy and Research Centre (ASTARC), Faculty of Medicine and Health Sciences, University of Antwerp, Antwerp, Belgium

**Keywords:** child, urinary incontinence, urotherapy tools, qualitative research, focus groups, patient experience, child-centered care, digital health, gamification

## Abstract

**Background:**

Standard urotherapy for childhood incontinence involves traditional tools like paper bladder diaries, timer watches, wetting alarms, and uroflowmeters. However, little is known about how these tools are experienced by today’s digitally native children.

**Objective:**

This study aimed to explore how children undergoing urotherapy perceive and experience these commonly used tools, with the goal of informing more engaging and child-centered design approaches.

**Methods:**

A qualitative focus group design was used with purposive sampling of children undergoing in-clinic urotherapy group training. In total, 19 participants (13 boys and 6 girls) aged 9-13 years took part in focus groups of 3 to 4 children, held at the hospital. A child-friendly focus group toolkit was used to facilitate discussion through creative and playful exercises. A total of 7 focus groups were conducted, including 2 repeated sessions, until thematic saturation was reached. All sessions were held in Dutch, video- and audio-recorded, and transcribed verbatim. An inductive conventional content analysis was conducted using a dual-coder approach to identify and iteratively refine emerging themes.

**Results:**

Four themes emerged: (1) attitudes and motivation: ranging from willingness to engage in urotherapy and use tools to reluctance or resistance; (2) social acceptance: highlighting the impact of peer perception, fear of being bullied, and opportunities to break the taboo and reframe tools as socially desirable; (3) contextual influences: including dissatisfaction with school toilets and limited child involvement at doctor visits, contrasted with the positive peer support experienced during group therapy; and (4) digital integration: children saw many traditional tools as outdated and suggested gamified, smart alternatives. The drawings created by children during the exercises served as a creative reflection of these thematic findings.

**Conclusions:**

Involving children in research and design is essential for creating interventions that are truly child-centered. Through creative, qualitative methods, this study uncovered rich insights into children’s experiences with urotherapy tools, pointing to 4 key design priorities: personalization, stigma-free design, adaptability, and digital innovation.

## Introduction

### Background

Daytime urinary incontinence affects many school-aged children (5‐13 y) [1,2], often leading to low self-esteem, feelings of shame, and stress, thereby negatively impacting the child’s quality of life [3,4]. Standard urotherapy is the recommended first-line treatment for daytime urinary incontinence [5]. During standard urotherapy, children receive age-appropriate information and instructions on drinking and toileting habits, which need to be repeatedly practiced in order to reach optimal results [6,7]. There are a variety of tools designed to support urotherapy, such as bladder diaries [8], timer watches [9], wetting alarms [7], and uroflowmeters [10].

Traditional bladder diaries consist of paper-based schedules where patients are required to manually record their urinary output (using a measuring cup), fluid intake, and incidents of urine leakage over a span of several days [1]. Bladder diaries provide the child, parent, and clinician with insights on symptoms and therapy progress and help to create self-awareness of voiding and drinking habits [5,11,12].

Timer watches sound an alarm at fixed time intervals to remind the child to drink and void regularly [9]. This tool encourages children not to postpone going to the toilet until they experience an urgent need or have accidents, but rather to urinate at set intervals with a more relaxed pelvic floor (ie,timed voiding).

Although primarily used for nighttime urinary incontinence, wetting alarms may also be used for children dealing with persistent daytime urinary incontinence [7]. These alarms involve specialized underwear with conductive material that triggers an alarm when wet. The purpose of the alarm is to restore a child’s bladder signal awareness and promote an appropriate response to it.

Finally, traditional uroflowmeters consist of a weight-based sensor with a urine receptacle on top, placed under a commode chair with funnel. They measure urine flow during urination and can be used for real-time biofeedback [13,14]. Through this feedback, children can better understand their voiding function and work toward normalizing their uroflow patterns.

### Study Rationale and Objectives

Despite its clinical potential, standard urotherapy is often challenged by low motivation and poor compliance [15]. Traditional tools may no longer align with the expectations of today’s digitally native children [16]. Yet, little is known about how these tools are perceived by children or how they influence their overall experience with urotherapy.

This study aims to gain a deeper understanding of the experiences, needs, and desires of pediatric patients regarding urotherapy and its tools. The ultimate goal is to inform the development of more engaging and child-centered interventions and tools that better align with their expectations.

## Methods

### Study Design

The study used a qualitative focus group research design, following the COREQ (Consolidated Criteria for Reporting Qualitative Research) guidelines. The focus group collected data on the experiences of pediatric patients with urotherapy and its tools.

### Participants and Recruitment

A purposive sampling strategy was used to recruit participants who were undergoing in-clinic urotherapy group training (voiding school) at the Wilhelmina Children’s Hospital of the University Medical Center Utrecht [17,18]. In this 10-day program, children are admitted to the hospital, staying overnight on weekdays and returning home over the weekend. The program includes a timed voiding and drinking schedule monitored through a bladder diary, along with the use of wetting alarms, repeated uroflowmetry biofeedback, and daily therapeutic sessions, all under the guidance of urotherapists.

All children who were enrolled in the program during the study period from February 2023 to April 2023 were considered eligible for participation in the focus group study. The inclusion and exclusion criteria were determined by the urotherapy program itself and included children aged 8 to 13 years with functional urinary incontinence that was refractory to standard urotherapy and who were fluent in Dutch. Children were excluded from the program if they had an anatomical or neurological cause for urinary incontinence, recurrent urinary tract infections, or unmanaged constipation. In addition, children were excluded if their cognitive functioning was below the level of a 6-year-old or if their IQ was below 60. These assessments were carried out by the clinical team of the Wilhelmina Children’s Hospital. During the first intake, an extensive psychosocial history was taken by the urotherapist, including school functioning. Children were expected to attend at least grade 3 of primary school in the Netherlands, be able to read, tell time, follow instructions, and attend either regular or special primary education. If there was any doubt about IQ or developmental delay, a psychological report was requested.

Information about the study was presented to both the child and a parent in written and verbal forms at the initiation of the urotherapy program, upon hospital admission. A total of 19 participants (13 boys and 6 girls), aged between 9 and 13 years (Table 1), participated in the study after parental informed consent was obtained. In addition, children aged 12 and older provided written consent, while younger participants gave assent. The children took part in focus groups of 3 to 4 participants, corresponding to the group they were assigned to within the urotherapy program. One child discontinued participation early due to difficulties with concentration and behavior that disrupted the group dynamics.

**Table 1.** Participant characteristics (N=19).

Characteristic	Participants, n (%)
Sex	
Male	13 (68)
Female	6 (32)
Age group (years)	
<10	5 (26)
10‐11	9 (47)
≥12	5 (26)
Diagnosis	
Overactive bladder	8 (42)
Voiding postponement	3 (16)
Dysfunctional voiding	2 (11)
Other	6 (32)
Comorbidities	
Neurodevelopmental or behavioral^a^	6 (32)
Other medical^b^	3 (16)
None	10 (53)

a Reported comorbidities included attention deficit disorder, attention deficit hyperactivity disorder, autism spectrum disorder, and dyslexia.

b Reported other medical comorbidities included hemophilia, leukemia and history of preterm birth.

### Ethical Considerations

The study was conducted in accordance with the Declaration of Helsinki. The Medical Ethics Review Committee NedMec reviewed the study protocol (22/955; decision date: November 15, 2022) and determined that it did not fall within the scope of the Dutch Medical Research Involving Human Subjects Act (WMO). This decision was based on the nonexperimental nature of the study, which involved no medical intervention and focused solely on exploring children’s experiences with existing urotherapy tools through focus group discussions. To ensure institutional oversight, the study was additionally reviewed and approved by the division management of the University Medical Center Utrecht.

Written informed consent was obtained from parents and from participants aged 12 years or older; younger participants provided assent. All data were pseudonymized before analysis. No compensation was provided for participation.

### Data Collection

All focus group sessions were conducted at the Wilhelmina Children’s Hospital during the in-clinic urotherapy program. The sessions were moderated by the first and third authors, both female researchers with no previous relationship to the participants. At the time of the study, the first author was a PhD candidate and the third author a master’s student at the University of Antwerp, Faculty of Design Sciences. The first author received formal training in qualitative research and was responsible for organizing and moderating the session. The third author supported the session through observation and note-taking.

At the start of each session, the researchers introduced themselves, explained their roles, and shared the purpose of the study. It was explained that the researchers were interested in designing better tools and services for children with urinary incontinence. The first author disclosed her background in pediatric health design research and her professional role in a urology-focused medical device company, acknowledging a personal and professional motivation to improve urotherapy experiences.

**Figure 1.**
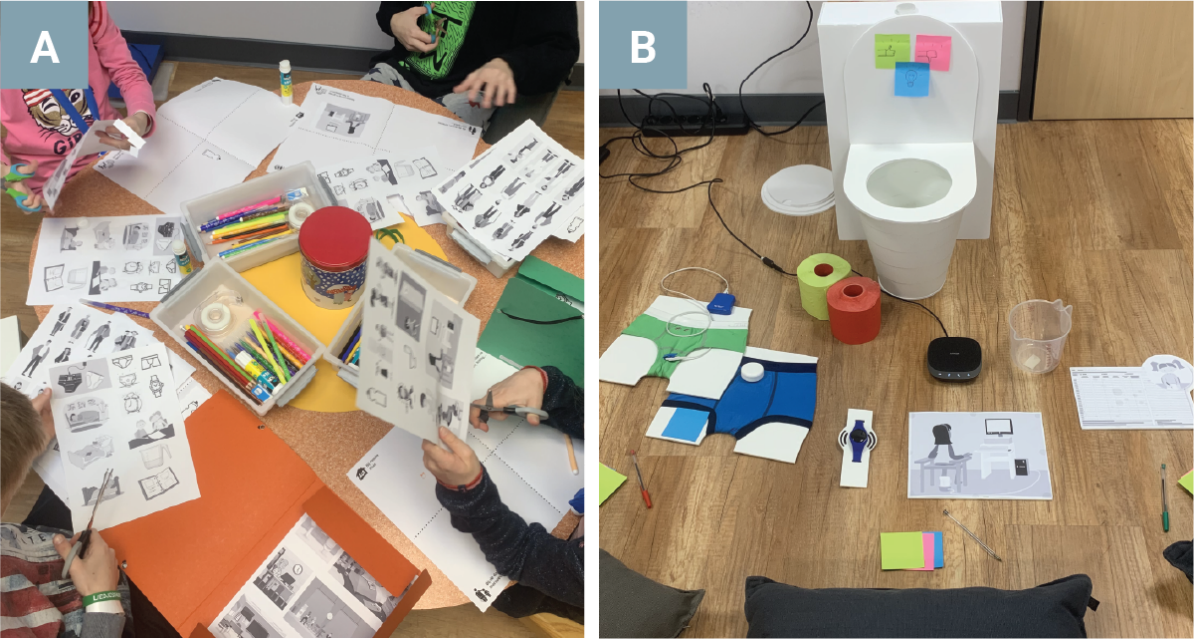
Child-friendly focus group discussion toolkit used in this study. (**A**) Collaging exercise where children shared emotions and personal experiences in various contexts. (**B**) Pen and paper exercise with cardboard toilet prop to share feedback and ideas on different urotherapy tools.

To support the children’s comfort at the start of the session, a urotherapist involved in their treatment was present. Once a safe and relaxed atmosphere was established, the urotherapist left the room, allowing the children to engage freely in the discussion with the researchers. Data collection was facilitated using a child-friendly focus group discussion toolkit, informed by existing literature and specifically designed for this study [19]. The goal was to create a safe and inspiring context where participating children could comfortably share personal experiences in a creative and playful manner (Figure 1). It consisted of an icebreaker and warm-up, collaging exercise, pen, and paper feedback, and a superhero character drawing (Table 2).

**Table 2.** Overview of focus group toolkit exercises.

Toolkit exercises	Description	Guiding questions
Icebreaker and warm-up	Children passed a toilet roll and shared personal facts. Followed by a group mind-mapping activity focused on drinking, going to the toilet, and bladder training.	“What is your name and age?,” “What are your hobbies?,” “When you hear the word ___, what comes to mind first?”
Collaging exercise	Children created collages about different settings (home, school, doctor visits, and group training) from the perspective of their bladder problems. They then presented their collages and discussed them in the group.	“What happens in those different contexts?,” “How do you feel in those contexts?,” “Who or what is there to support you?”
Pen and paper feedback	Children reflected on urotherapy tools (bladder diaries, timer watches, wetting alarms, and uroflowmeters) by drawing or writing their thoughts on paper. These were thrown into a cardboard toilet prop and then randomly drawn to prompt group discussion.	“What do you like and don’t like about this tool?,” “What ideas do you have to make it better?”
Superhero character drawing	Children drew a superhero who, like them, also happens to have a bladder problem. They then presented their superheroes and described their powers and coping strategies.	“What is your superhero’s name?,” “What superpowers do they have?,” “How do they deal with their bladder problems?”

Each session lasted between 1.5 and 2 hours. Data collection concluded upon reaching “thematic saturation” (ie, when no new themes are uncovered by additional sessions) [20]. This saturation point was achieved after 7 focus groups, including 2 repeated sessions where 2 groups participated in an additional session on another day. These follow-up sessions were conducted to explore certain themes in more depth.

All focus group sessions were conducted in Dutch, video and audio recorded, and transcribed verbatim. The excerpts quoted in this paper were translated into English by the first author, with the aim of preserving the original meaning and tone. Due to the young age of participants, transcripts were not returned, and member checking was not conducted. However, to enhance the credibility and trustworthiness of the findings, they were cross-checked with health care professionals involved in the treatment of the participating children.

### Data Analysis

A conventional content analysis [21] was used to inductively derive meaning from the focus group data, following three main phases: preparation, organization (including coding), and reporting.

In the preparation phase, the first author repeatedly watched the video recordings and read the transcripts to gain a comprehensive understanding. This phase allowed the researcher to become familiar with the content and begin identifying meaningful units (segments of the text) of analysis.

In the organization phase, relevant segments of text were manually highlighted and open-coded using Microsoft Excel. Through several iterations of coding and code modification, a codebook was developed. The transcripts were then further coded by pairs of master’s students, using a dual-coder approach to ensure consistency. As coding progressed, subcategories emerged and were iteratively grouped into broader themes, capturing patterns across the dataset. This hierarchical structure of codes, subcategories, and themes constituted the coding tree developed during analysis.

In the reporting phase, the first author prepared the preliminary structure of the emerging themes, which were then discussed and validated within the multidisciplinary research team, including a pediatric urologist, 2 urotherapists, and 2 medical product designers. This collaborative step ensured a diversity of viewpoints and enhanced the trustworthiness of the analysis. The final themes were defined based on consensus within the team and formed the basis for the study’s results.

## Results

### Overview of Themes

Four main themes emerged from the analysis that provide insight into how children perceive and engage with urotherapy and its tools: (1) attitudes and motivation (2) social acceptance, (3) contextual influences, and (4) digital integration. The structure of the themes and subthemes is shown in Textbox 1.

Textbox 1. **Theme structure.**

**Attitudes and motivation**

- Willingness to engage in training and use tools
- Resistance or ignoring support
- Parental involvement and tension
- Fear of dependence and desire for autonomy

**Social acceptance**

- Need for discreet design
- Fear of being bullied
- Openness as a way to break taboo
- Reframing tools as socially desirable

**Contextual influences**

- Bladder behavior varies by setting
- Dissatisfaction with school toilets
- Disconnection at the doctor’s office
- Peer support during group urotherapy

**Digital integration**

- Children are digital natives
- Traditional tools seen as outdated
- Ideas for gamified, smart solutions
- Awareness of digital pitfalls

### Theme 1: Attitudes and Motivation

Children expressed diverse attitudes and varying levels of motivation toward engaging with urotherapy and its tools. For instance, a clear willingness to participate in training emerged, along with recognition of the value urotherapy tools could offer in managing their condition. Tools were described as useful for reminding them to go to the toilet, increasing awareness of bladder signals, and providing objective data to better understand their condition.

If your mom or dad don’t remind you to go to the toilet, then you have to do it yourself. That’s when the timer watch is really helpful.

[P3, male, 10 y]

When you wear the wetting alarm, you can actually feel what it’s like when you leak. When it starts beeping, you know what that feeling means. Without it, you don’t really know what to pay attention to.

[P11, male, 10 y]

A bladder diary helps you see how much you pee. If you just go to the toilet normally, you can guess, but you don’t really know. But with a measuring cup, you know exactly. If it’s very high or very low, the doctors can figure out if something’s wrong or not, and then they can help you.

[P12, female, 9 y]

In contrast, expressions of reluctance or resistance were also observed. This included deliberately ignoring advice or recommendations like pretending to go to the toilet, disregarding alerts from reminder tools, or even ignoring their own bladder signals in favor of continuing preferred activities.

You can fake going to the toilet. Just go to the bathroom without actually peeing, and then come back.

[P3, male, 10 y]

I didn’t always go to the toilet when the timer watch went off. Sometimes I just didn’t feel like it and kept playing. I just turn off the alarm.

[P7, male, 8 y]

I’m pretty stubborn. I usually ignore my bladder signal. Even if an alarm goes off, I ignore it because I really want to finish my game. And then… well, I usually end up wet.

[P11, male, 10 y]

Children reported that in these situations parents became more involved in supporting their urotherapy routines. This involvement sometimes led to feelings of tension, frustration, or being controlled. The use of certain tools to enforce behavior was mentioned as a potential trigger for arguments between children and parents.

After I turned off the timer watch, my mom reprogrammed it to go off every thirty minutes and she turned up the volume so my teacher would hear it too and make sure I actually went to the bathroom.

[P1, male, 9 y]

Keeping a bladder diary at home is so annoying. Like, if you’re mad at your mom and you need to go to the bathroom, you still have to fill it in, and then she’ll yell, “Fill it in!”

[P13, female, 10 y]

My mom looks at my uroflow curves and then complains, like, “Such small voids!”

[P14, female, 13 y]

In addition, children expressed concerns about becoming dependent on certain urotherapy tools, emphasizing their desire for autonomy. They discussed how rigid, time-based routines set by tools like timer watches do not always align with real-life situations. They also described these tools as temporary aids to support their progress toward independent bladder control.

I don’t like the timer watch because it makes you depend on it. When it beeps, you have to go, but what if you’re busy and can actually hold it? Or what if you just don’t need to go?

[P16, male, 12 y]

You don’t have a uroflow toilet at home, so you can’t really see how you pee. But in the end, you should be able to do it without one.

[P11, male, 10 y]

### Theme 2: Social Acceptance

Social acceptance emerged as a significant theme, reflected in the importance participants placed on how they are perceived by peers. A recurring concern was that urotherapy tools were not discreet and could attract unwanted attention in social settings, leaving children feeling singled out.

If you’re outside and the timer watch starts beeping every hour, your friends are like, “Why does he keep beeping?”

[P2, male, 11 y]

I had to keep a bladder diary at school once, but it was so annoying. Everyone in the class would look at me when I had to do it.

[P14, female, 13 y]

If you wear the alarm around your neck, everyone knows you’re doing bladder training.

[P11, male, 10 y]

In addition, children reported being bullied for their bladder problems. To cope with this, they described efforts to hide symptoms and to be very selective about who they trusted with information about their condition.

I had an argument with my little brother and then I wet my pants, which happens more easily when I’m stressed. And then he yelled “Ew, gross! Run away! Run away!”

[P8, male, 11 y]

Only my teachers know. I don’t tell anyone else, because I know I’ll get bullied again. Like once during gym class, someone saw I had a wet spot. But I grabbed a glass of water and pretended to spill it on myself so they’d think it was just water.

[P2, male, 11 y]

In contrast, children expressed that openness about their bladder problems could serve as a way to break the taboo. For those who chose to do so, this openness was met with peer support and contributed to greater self-acceptance.

I did my school presentation about my bladder problem. That really helped me, because now my classmates support me. I also have a really good best friend who visits me a lot, and we often talk about it.

[P4, male, 9 y]

I think it’s important that you can still fit in at school, even with this problem. Almost my whole class knows about it.

[P3, male, 10 y]

Timer watches were not always associated with stigma. Children described that, when designed in a playful, everyday style, the watches could attract desired attention. In these cases, the timer watch was seen as a stigma-free and unique accessory that could be proudly shown to peers.

I had one of those timer watches once in rainbow colors, and some kids said, “Wow you have a really cool watch!”

[P3, male, 10 y]

When the other kids in the class don’t have a watch, they get really jealous.

[P4, male, 9 y]

### Theme 3: Contextual Influences

**Figure 2.**
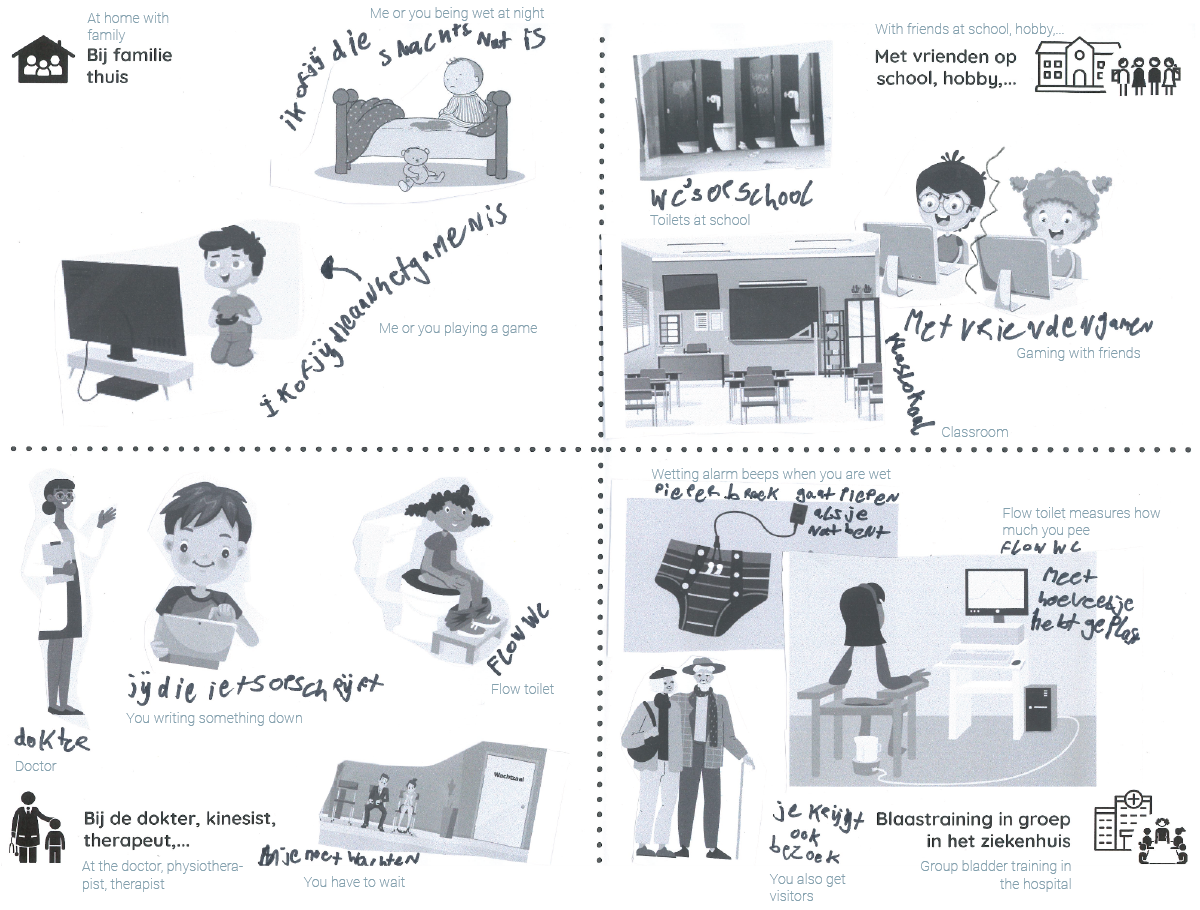
Example of a collage created by a participant (gray text represents the English translation).

The collaging exercise in particular revealed how various contexts influence children’s bladder behavior (Figure 2). Different contexts, such as home, school, or sports, led to varying patterns of hydration, awareness of bladder signals, and incontinence frequency. Gaming and sports were identified as contexts where urine leaks occurred more frequently due to distraction, while school environments were associated with greater attentiveness and fewer incidents.

At home I game a lot, and then it sometimes goes wrong. I get wet. Because while gaming, I don’t really feel my signals. At school it goes better, because I pay attention to my bladder. I also drink less at home.

[P11, male, 10 y]

I swear, when I get wet, it’s usually during sports. It just leaks without me noticing.

[P2, male, 11 y]

At school or with my friends, I drink a lot and then I go to the bathroom quickly. At home, I barely drink anything. I have to manage everything myself there, and then I forget.

[P5, female, 9 y]

At school, I don’t really drink anything, so I don’t have to pee.

[P15, female, 12 y]

Furthermore, specific contexts such as school toilets present their own unique challenges. Dissatisfaction with school toilets was a recurring theme, with children highlighting issues related to poor hygiene and restrictive policies.

At our school, the toilets smell really bad.

[P15, female, 12 y]

The toilets at school are really, extremely gross.

[P11, male, 10 y]

I got punished at school because I had to go to the toilet.

[P8, male, 11 y]

I can go to the toilet at school whenever. Only sometimes they lock the entrance door.

[P11, male, 10 y]

Similarly, the doctor’s office presented its own challenges as a context. Children reported feeling disconnected during consultations, as interactions were often directed more toward their parents. In these situations, they described becoming disinterested, confused, or disengaged.

The doctor mostly talks to my mom. I just start playing with something, because I don’t understand everything they’re saying. Then my mom gets mad and says, “Hey, you have to pay attention!”

[P11, male, 10 y]

We sometimes go to a doctor, but I don’t really know what he does.

[P6, female, 9 y]

When I’m at the doctor’s office, I go to the toilet just because I don’t like being there.

[P13, female, 10 y]

In contrast, children described their experiences with in-clinic group therapy very positively. A key factor mentioned by children was the peer support they received from others going through similar challenges. Being part of a group was described as giving them a sense of belonging, trust, and shared understanding, which contrasted with the feelings of isolation often experienced at home or in other settings.

I really looked forward to it. It’s actually like a sleepover. You can talk to each other 24/7. And it’s just nice to be in a room together, play a game, or go gaming together. You sit next to each other, so you can see what’s happening. At home, you’re really just doing everything on your own.

[P11, male, 10 y]

It’s just nice when someone you really trust and like has the same problem as you.

[P3, male, 10 y]

### Theme 4: Digital Integration

As digital natives, the children are highly familiar with digital technologies. This was reflected in the focus groups through references to the regular use of smartphones, games, and other digital technologies.

Well, that’s literally who I am: phone, selfies, calling.

[P15, female, 12 y]

At first, I only had two and a half hours of screen time on my phone, but now I’m just allowed to use it as much as I want.

[P17, male, 12 y]

At home I game a lot. Gaming is my hobby.

[P11, male, 10 y]

Everything is digital these days.

[P4, male, 9 y]

I’m not much of a writer.

[P14, female, 13 y]

In discussing urotherapy tools, children referred to their familiarity with digital platforms and contrasted this with traditional tools, such as paper-based bladder diaries or longstanding tools like wetting alarms and timer watches. They reported that these traditional tools were outdated, inconvenient, and misaligned with their digital preferences.

The wetting alarm and timer watch are old-fashioned!

[P9, male, 9 y]

It’s annoying that you have to write everything down in your bladder diary. You just want to get on with what you’re doing.

[P11, male, 10 y]

I’m okay with using the measuring cup, but not the paper chart. It’s just inconvenient.

[P12, female, 9 y]

When the wetting alarm goes off, it just vibrates or beeps, but that’s kind of basic and just makes you nervous. It should go off in some other, more fun way.

[P3, male, 10 y]

Children also came up with creative suggestions for smart, gamified solutions to enhance the appeal, engagement, and usability of urotherapy tools. These ideas included digitizing traditional tools such as bladder diaries.

The bladder diary should be digital so it’s quicker to use.

[P3, male, 10 y]

There should be something that fills in the bladder diary automatically.

[P13, female, 10 y]

The timer watch should also let you record when you’ve been wet, like a bladder diary.

[P3, male, 10 y]

Among the ideas expressed were advanced features, such as smart algorithms capable of predicting when the bladder is full or when it is time to drink. It was also suggested that these systems could include self-learning capabilities, adapting over time based on user behavior and feedback.

Something that beeps when your bladder is full, instead of when you’re already wet.

[P8, male, 11 y]

Something that predicts when you need to drink and pee using sensors that track how much you’ve drunk over time, and then tells you when you’ll need to pee. You can see it in an app, and maybe even give feedback on whether the prediction was right or not.

[P16, male, 12 y]

Gamification also emerged as a strategy to increase engagement, with ideas such as turning uroflowmetry into a game through scoring systems that reward performance and progress.

There should be a mini game on the uroflow toilet. One time I set the record. I think it was 330 ml.

[P10, male, 10 y]

You could get a score for your uroflow graph, for example, 25 points for “Nice,” 50 for “Good,” 75 for “Perfect,” and 100 for “Excellent.” But it’s important that the points always stay positive.

[P19, male, 13 y]

**Figure 3.**
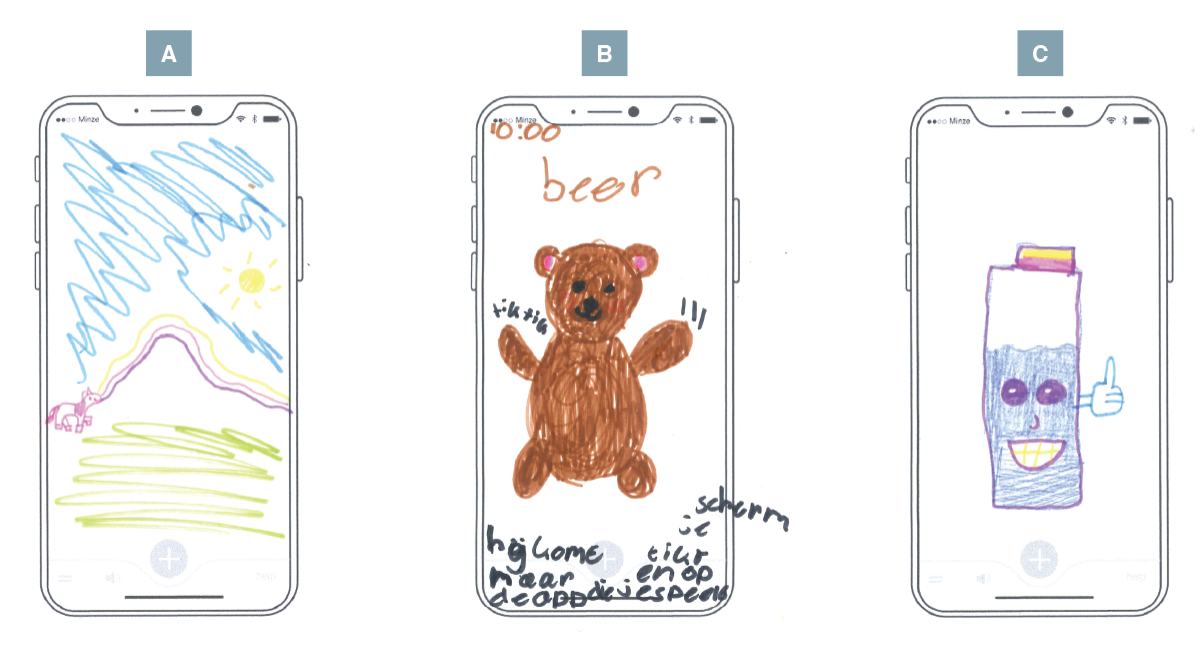
Children’s drawings illustrating concepts for a digital training buddy. (**A**) Little animal that walks across your uroflow curve; (**B**) little bear that reminds you to go to the toilet; (**C**) bottle that gets happier the fuller it gets to remind you to drink.

Finally, an interactive training buddy, such as a virtual animal or bottle avatar (Figure 3), was proposed as a way to provide age-appropriate encouragement and a sense of companionship.

There could be a little animal, one you get to choose, that walks across your uroflow curve.

[P6, female, 9 y]

If you haven’t peed in a while, a little bear shows up in the game you’re playing on your phone, taps the screen, and says, “You have to go to the toilet!”

[P13, female, 10 y]

To help you drink more, there’s a bottle on your phone that fills up and gets happier the fuller it gets. When it’s completely full, it looks really happy.

[P6, female, 9 y]

Despite enthusiasm for digital solutions, children also raised concerns. These included excessive phone use, technical malfunctions, or limitations, and the risk of fostering an overly competitive mindset through gamified features, which could result in disappointment.

If I start doing the bladder diary on my phone, I’ll end up looking at other stuff and three hours later, I’m still on my phone.

[P14, female, 13 y]

If there’s a technical error, your data might not get saved.

[P19, male, 13 y]

If you’re out and forget your phone, and that’s where the bladder diary app is, then you’d have to go back and get your phone.

[P18, male, 11 y]

If you make it into a game, then you really want to win, but if you don’t succeed, you might feel embarrassed or disappointed, and that’s not really nice.

[P11, male, 10 y]

### Thematic Reflections in Children’s Superhero Drawings

The superhero drawing exercise served as a creative and expressive validation of the findings from the focus groups, visually reflecting the core themes that emerged throughout the study. The characters children designed included elements that represented their attitudes, experiences, and desires.

**Figure 4.**
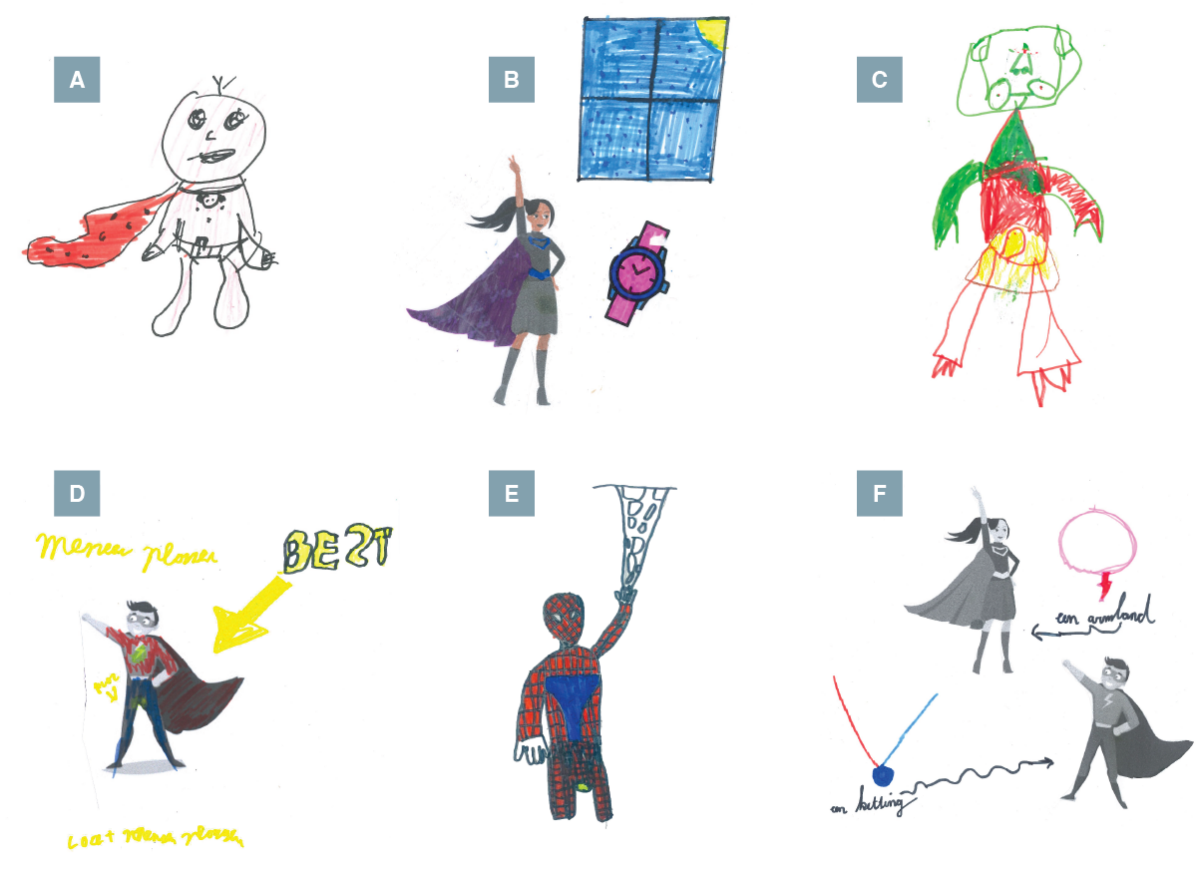
Children’s superhero drawings. (**A**) “Captain Underpants” with wetting alarm; (**B**) “Time Controller” with timer watch to master toilet timing; (**C**) “Diaperman” with an infinite diaper supply; (**D**) “Mister Pee” with the power to make bullies wet their pants; (**E**) Spiderman with a wet spot; (**F**) Different superhero accessories to prevent urination while wearing them.

The theme of attitudes and motivation included representations of superheroes who showed a willingness to participate in training and make use of urotherapy tools. For example, child 3 (male, 10 y) created “Captain Underpants,” a popular Dutch children’s book character, with a wetting alarm attached to his underpants (Figure 4A). Similarly, child 14 (female, 13 y) illustrated the “Time Controller,” using tools like the timer watch to master toilet timing (Figure 4B). Child 2 (male, 11 y) envisioned a well-trained superhero with a powerful metal sphincter to prevent urine leakage.

In contrast, child 10 (male, 10 y) created “Diaperman,” a robot with an infinite diaper supply (Figure 4C), and child 4 (male, 9 y) a superhero with laser eyes to remove the bladder. These superheroes may reflect a desire for convenience or unrealistic quick fixes rather than commitment to training, aligning with the subtheme of resistance toward training under the same theme of attitudes and motivation.

The theme of social acceptance also emerged through characters such as “Mister Pee” (child 8, male, 11 y), whose superpower is making bullies wet their pants so they can understand what it feels like (Figure 4D), an embodiment of the fear of being bullied. In contrast, characters like Spiderman with a wet spot (Figure 4E) or Superman with a diaper (child 11, male, 10 y) reinforced the idea that bladder problems can affect anyone, even strong and popular superheroes, supporting the subtheme of openness as a way to break the taboo.

Child 12 (female, 9 y) designed different superhero accessories that grant the power to completely stop urination, but only when worn (Figure 4F). This drawing was categorized within the theme of contextual influences, as it illustrated how bladder control was imagined as dependent on specific situations or conditions.

Finally, many superheroes across drawings were equipped with tools and gadgets, hinting at the theme of digital integration, particularly the subtheme of gamified and smart solutions. These imaginative tools and gadgets often combined functionality with fantasy.

## Discussion

### Principal Findings

This qualitative focus group study revealed that children’s experiences with urotherapy and its tools are shaped by a combination of (1) attitudes and motivations, (2) the need for social acceptance, (3) contextual influences, and (4) a strong desire for digital integration. As digital natives, children perceived many traditional urotherapy tools as outdated and misaligned with their expectations. They expressed a need for tools that support autonomy, discretion, and adaptability. Tools designed in a playful, everyday style were considered less stigmatizing and more socially acceptable. Their own innovative suggestions, ranging from smart predictive algorithms to gamified platforms and interactive training buddies, reflect a desire for more advanced, technology-driven solutions. At the same time, they acknowledged potential pitfalls, such as overuse or dependence on technology.

### Strengths and Limitations

We conducted a qualitative study using a novel focus group discussion toolkit comprised of creative and playful exercises tailored to our objectives. While qualitative research in pediatric urology is an emerging field, its potential to capture the lived experiences of young patients is increasingly recognized [22]. The creative exercises used in this study enabled children to share personal and sensitive experiences in a child-friendly and expressive manner, demonstrating the potential of creative methodologies to enrich data quality and participant engagement. To our knowledge, no previous study has adopted such an approach in this field.

This study also has limitations. First, the focus group sessions were relatively long (1.5 to 2 h), which posed challenges in maintaining participants’ attention and cooperation. Nevertheless, the variety of creative and engaging exercises helped sustain children’s attention. Second, the generalizability of findings is limited due to the purposive sampling of children undergoing the most intensive form of urotherapy, in-clinic group training in a tertiary care center. Despite this, we believe some transferability exists. These participants can be considered experts by experience, having undergone a broad spectrum of care ranging from outpatient to inpatient urotherapy. Moreover, their strong interest in advanced digital solutions likely reflects a broader trend among children growing up in a digitally native generation. Therefore, while findings should be interpreted within context, they provide valuable insights for improving urotherapy practices and inspiring the design of future urotherapy tools, particularly those involving smart, digital innovations.

### Comparison With Prior Work

Our findings align with previous research demonstrating the social impact of peer dynamics, including a desire to conceal bladder problems and fear of bullying [23,24]. Similarly, our results are consistent with earlier studies highlighting contextual challenges, such as dissatisfaction with school toilets [25,26] and limited involvement during doctor consultations, in contrast to the positive influence of peer support experienced during group therapy [27]. Finally, children’s interest in gamified, smart solutions aligns with the findings of our concurrent scoping review on innovative, technology-driven, digital tools for managing pediatric urinary incontinence [28]. Many of the tools identified in this review were described as engaging for children, improving patient compliance and leading to high levels of patient satisfaction and preference. Their gamification ideas aligned with the serious games described in the review, further validating the appeal of playful, story-driven engagement strategies. Similarly, the concept of prevoid alarms, which featured prominently in the review, was also proposed by children.

Both the scoping review and the focus group point to critical design features for future product development, including adaptability, personalization, and smart connected systems. What the focus group adds, however, is an important user-centered depth. To truly personalize treatment or tools, one must understand the child’s individual context: attitudes, motivation, social context, and emotional needs. Yet, as the focus group revealed, children are not always actively involved during medical consultations, raising the question of how personalization can occur without their active participation. Importantly, the focus group study demonstrated that children can articulate their needs and preferences, especially when supported by creative, child-friendly methods. Their creative input provided a valuable entry point into co-design and communication strategies, as explored further in the Implications and Future Directions section.

### Implications and Future Directions

**Figure 5.**
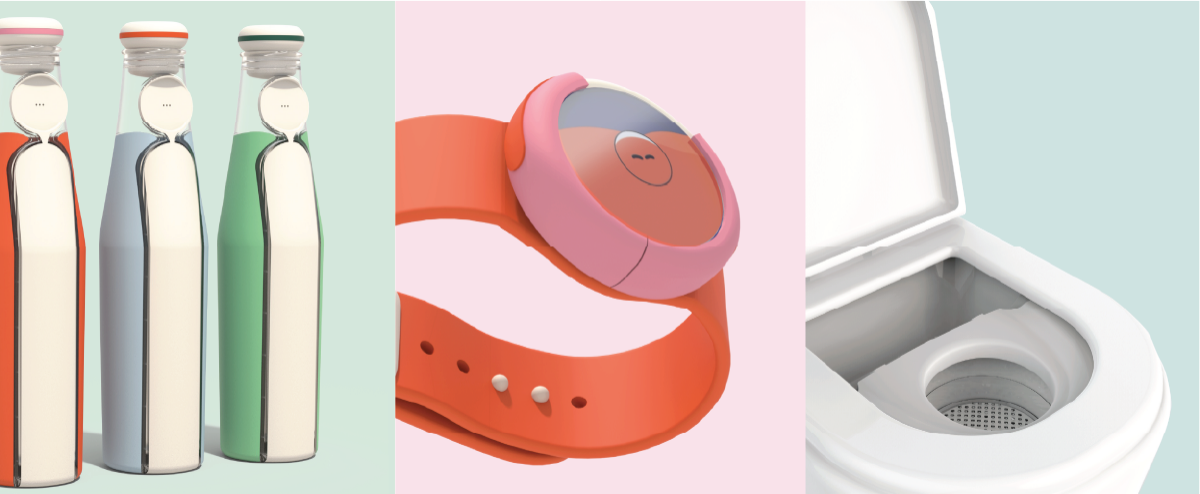
Smart urotherapy concept: a smart drinking bottle, reminder watch, and home-based uroflowmeter are interconnected to deliver personalized voiding and drinking prompts via a digital companion on the watch.

Building on the children’s creative ideas shared during the focus groups, we designed a conceptual smart urotherapy solution [29]. This concept incorporates smart algorithms capable of predicting bladder fullness and optimal drinking times by connecting a smart drinking bottle with a home-based uroflowmeter (Figure 5). These devices are linked through a reminder watch, which offers personalized voiding prompts based on the child’s fluid intake and estimated bladder capacity. To reduce stigma and enhance social appeal, the design shifts the focus from urination to drinking and features playful, child-friendly esthetics. A digital training buddy, displaying various hydration and urgency states, provides companionship, support, and motivation throughout the therapy process.

**Figure 6.**
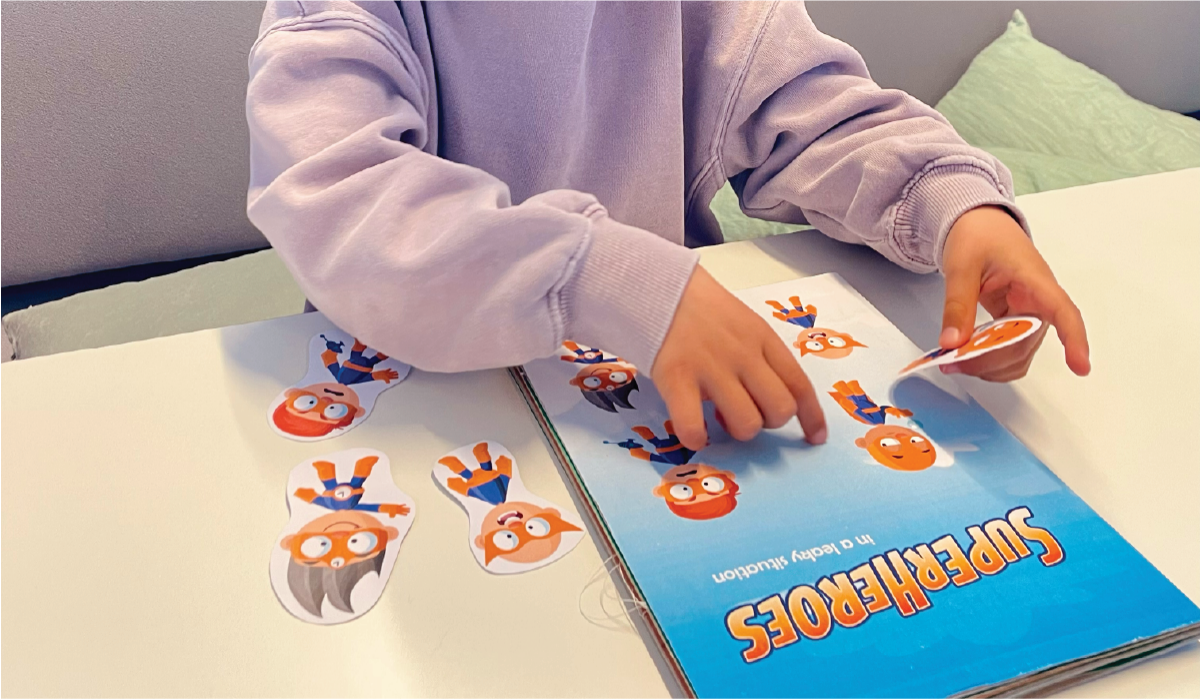
A first design of a urotherapy superhero family, intended as a conversation tool between children and health care providers to promote engagement and a greater sense of child involvement.

Findings also highlight the need for improved child involvement during doctor visits. Children often lacked awareness of their care pathway, suggesting a need for more child-centered communication. The creative methods used, particularly the superhero drawing activity, proved effective in facilitating meaningful dialog and capturing children’s emotions, motivations, and experiences. These insights have informed follow-up research focused on further analyzing the drawings to develop a “urotherapy superhero family,” intended as a conversation tool during consultations (Figure 6). By inviting children to select a hero companion that represents them during treatment, the tool aims to enhance child engagement, support emotional expression, and promote a more active role in their care trajectory.

### Conclusions

This study’s use of qualitative and creative methods enabled a rich understanding of how children engage with urotherapy and the tools that support it. It revealed that their experiences are shaped by a complex interplay of personal attitudes and motivations, social dynamics, contextual factors, and a strong desire for digital integration. These insights offer direction for developing future tools that prioritize personalization, stigma-free design, adaptability, and digital innovation. Including children’s voices in research and design is essential to ensure that interventions are not only clinically effective but also meaningful and engaging, ultimately contributing to more child-centered care.

## Supplementary material

10.2196/79142 Checklist 1 COREQ checklist.

## Abbreviations

COREQ: Consolidated Criteria for Reporting Qualitative Research
